# The application of CRISPR/Cas technologies to *Brassica* crops: current progress and future perspectives

**DOI:** 10.1007/s42994-022-00076-3

**Published:** 2022-07-02

**Authors:** Jun Li, Xiaoxiao Yu, Chao Zhang, Na Li, Jianjun Zhao

**Affiliations:** 1 State Key Laboratory of North China Crop Improvement and Regulation, College of Life Sciences, Hebei Agricultural University, Baoding, 071001 China; 2 Key Laboratory of Vegetable Germplasm Innovation and Utilization of Hebei, Collaborative Innovation Center of Vegetable Industry in Hebei, College of Horticulture, Hebei Agricultural University, Baoding, 071001 China

**Keywords:** CRISPR, Cas9, Cas12, Precise genome editing, *Brassica*

## Abstract

*Brassica* species are a global source of nutrients and edible vegetable oil for humans. However, all commercially important *Brassica* crops underwent a whole-genome triplication event, hindering the development of functional genomics and breeding programs. Fortunately, clustered regularly interspaced short palindromic repeat (CRISPR)/CRISPR-associated (Cas) technologies, by allowing multiplex and precise genome engineering, have become valuable genome-editing tools and opened up new avenues for biotechnology. Here, we review current progress in the use of CRISPR/Cas technologies with an emphasis on the latest breakthroughs in precise genome editing. We also summarize the application of CRISPR/Cas technologies to *Brassica* crops for trait improvements. Finally, we discuss the challenges and future directions of these technologies for comprehensive application in *Brassica* crops. Ongoing advancement in CRISPR/Cas technologies, in combination with other achievements, will play a significant role in the genetic improvement and molecular breeding of *Brassica* crops.

## Introduction

The genus *Brassica*, belonging to the family Brassicaceae, includes many economically valuable crops that are used as vegetables, oilseeds, and condiments worldwide (Chen et al. 2019a). Six crop species are of particular agricultural importance, and their evolutionary relationships are described by U’s triangle. Three of these species are diploid [*Brassica rapa* (AA), *Brassica nigra* (BB), and *Brassica oleracea* (CC)], while the other three are allotetraploids [*Brassica napus* (AACC), *Brassica juncea* (AABB), and *Brassica carinata* (BBCC)] derived from each pair of the three diploid species. All six *Brassica* species underwent a recent whole-genome triplication event, resulting in a high number of duplicated genes (Wang et al. 2011). The complex polyploid nature of *Brassica* has hindered the development of functional genomics and breeding programs.

Traditional breeding, molecular marker-assisted selection breeding, and transgenic breeding have been used in *Brassica*; however, each of them has restrictions or shortcomings (Chen et al. 2019b). Thus, there is significant need to introduce new plant breeding technologies to accelerate germplasm innovation. CRISPR/Cas technology, which allows the editing or modulation of DNA sequences within an endogenous genome, is the most widely used genome-editing technologies (Gao et al. 2021). Considering that single or multiple nucleotide substitutions are crucial for crop improvements (Mao et al. 2019), precise genome-editing platforms based on CRISPR/Cas are highly valuable and have evolved rapidly. These CRISPR/Cas technologies (e.g., base editing, prime editing) have been successfully applied to a broad range of plant species. However, it is predominantly exploited to knock out genes using CRISPR/Cas9 in *Brassica* species, there is significant room for improvement.

Herein, we provide a brief overview of current CRISPR/Cas technologies, including CRISPR/Cas9 and CRISPR/Cas12, and we summarize existing engineered or evolved Cas9 and Cas12a variants with broadened targeting ranges and improved editing specificity. Next, we discuss technical breakthroughs based on CRISPR (e.g., base editing and prime editing), which can carry out precise genome modifications. We also review recent progress in the application of CRISPR/Cas technologies to *Brassica* species. Finally, current challenges and future perspectives on the use of CRISPR/Cas technologies for *Brassica* improvement are discussed.

## Recent progress in CRISPR/Cas technologies

**Fig. 1:**
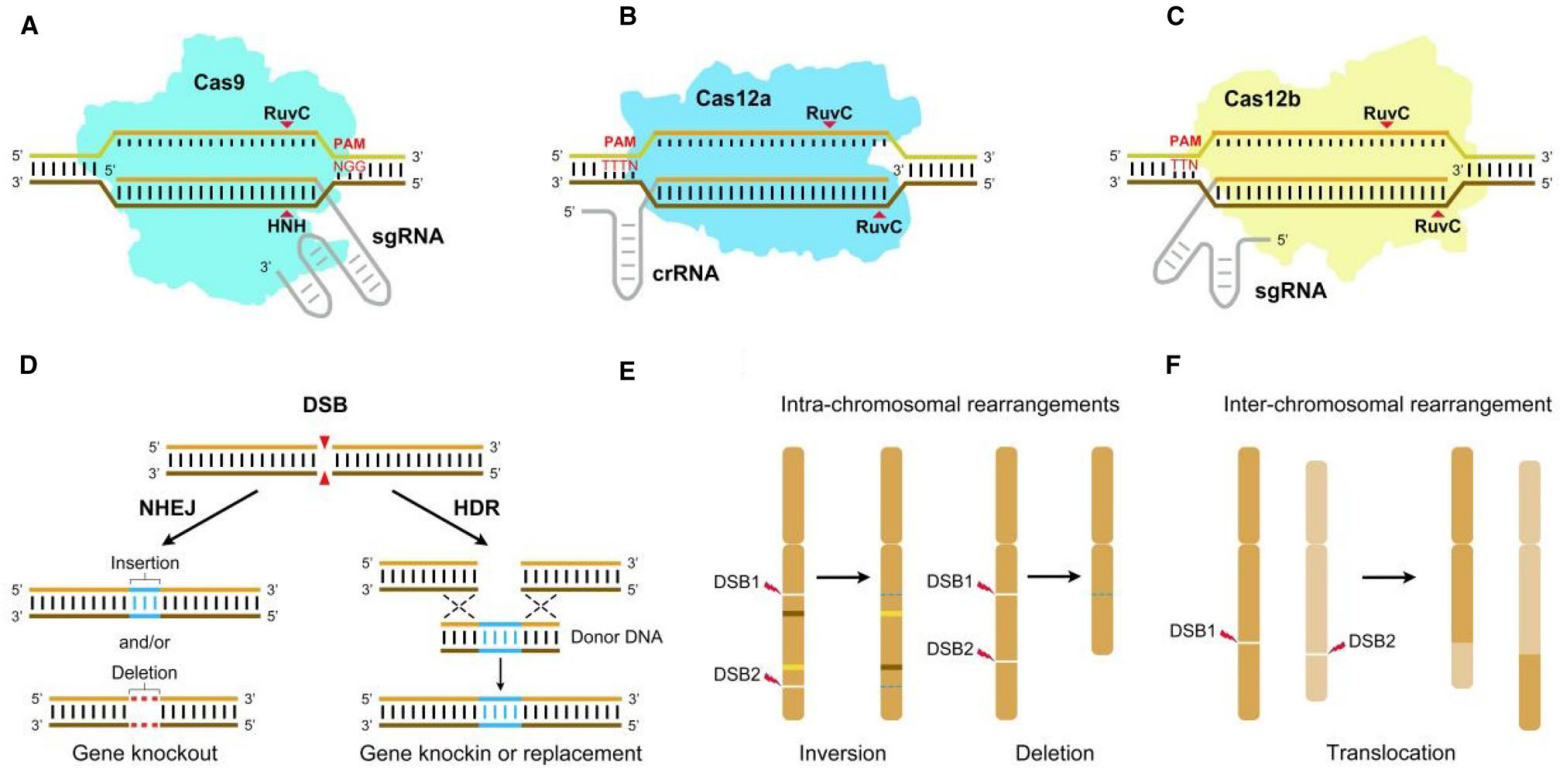
Overview of the main CRISPR/Cas systems used for genome editing and the repair of DSBs. **A** CRISPR/Cas9. Guided by the sequences on the sgRNA and PAM downstream of the target, Cas9 cleaves the double-stranded DNA, creating a blunt-ended DSB. **B** CRISPR/Cas12a. Guided by the sequences on the crRNA and PAM upstream of the target, Cas12a cleaves the targeted DNA, generating a staggered DSB. **C** CRISPR/Cas12b. Guided by the sequences on the sgRNA and PAM upstream of the target, Cas12b cleaves the targeted DNA, generating a staggered DSB. **D** A single DSB is repaired via the NHEJ or HDR pathway. In the NHEJ pathway, there are variable insertions (blue line) and/or deletions (red dotted line) at the break, generating a knockout mutant. When there is a donor template around the DSB, it can be repaired via the HDR pathway, generating predefined, precisely repaired mutants. **E** Two DSBs in the same chromosome can induce intra-chromosomal rearrangements, leaving indels (blue dashes) at the break. **F** Two DSBs in different chromosomes can induce inter-chromosomal rearrangement

CRISPR/Cas is a prokaryotic adaptive defense system used to fight off invading genetic materials (viruses or plasmids) in bacteria and archaea (Chen et al. 2019b). CRISPR/Cas systems evolve rapidly, leading to extreme structural and functional diversity. Based on the conservation and locus organization of Cas, the systems are divided into two classes: class 1 (types I, III, and IV) and class 2 (types II, V, and VI). With breakthroughs in understanding the defensive processes of CRISPR/Cas systems, CRISPR/Cas9 and CRISPR/Cas12 have been exploited as RNA-programmable genome-editing tools (Fig. 1A–C). Due to their simplicity, high efficiency, versatility, and capacity for multiplexing, CRISPR/Cas technologies have been widely used and have revolutionized all areas of molecular biology (Anzalone et al. 2020; Gao 2021).

### Mechanisms of CRISPR/Cas-mediated genome editing

Relying on DNA–RNA recognition and binding for targeted DNA breaks, CRISPR/Cas systems are programmed to genome-editing tools (Jinek et al. 2012). These are used to induce site-specific double-strand breaks (DSBs) in the targeted genomic sequence. Once the DSB is made, it triggers two main endogenous DNA repair pathways: non-homologous end joining (NHEJ) and homology-directed repair (HDR) (Fig. 1D). NHEJ is an error-prone pathway. When a DSB is repaired by NHEJ, two broken ends are simply religated, generating uncontrolled insertions/deletions (indels) at the junction of the rejoined chromosome. When pairs of DSBs are created simultaneously, targeted chromosomal rearrangements (e.g., deletions, inversions, and translocations) can be generated between the two breaks (Fig. 1E, F). HDR is a high-fidelity repair process. If a homologous template is provided, HDR may occur, generating precise genome edits, including point mutations, insertions, replacements, and deletions. However, the efficiency of HDR is extremely low in plant cells (Chen et al. 2019b; Mao et al. 2019).

### CRISPR/Cas9

Cas9 from the class 2 type II CRISPR system is an RNA-guided endonuclease. In nature, CRISPR RNA (crRNA) and trans-activating crRNA (tracrRNA) form a two-RNA structure that directs Cas9 to introduce DSB in the target DNA. However, the most widely used CRISPR/Cas9 technology relies on a single guide RNA (sgRNA) engineered from the dual-tracrRNA:crRNA (Jinek et al. 2012). Guided by the target sequence within the sgRNA, Cas9 creates a blunt-ended DSB about 3 bp upstream of the protospacer adjacent motif (PAM, NGG) (Fig. 1A).

Since the first report of programmed DNA cleavage by Cas9 from *Streptococcus pyogenes* (SpCas9) in vitro, Cas9 orthologs have been discovered and tested for genome editing (Gürel et al. 2020). These Cas9 endonucleases differ mainly in their overall size, PAM sequence, guide RNA architecture, and editing efficiency. This has expanded the CRISPR toolbox for genome editing. Due to the high efficiency and versatility, CRISPR/Cas9 has proven to be the best choice for genome editing in numerous species (Chen et al. 2019b; Mao et al. 2019).

### CRISPR/Cas12

Cas12, another RNA-guided endonuclease from the class 2 type V CRISPR system, has also been explored. In nature, many Cas12 endonucleases are guided by a single crRNA while some use an additional tracrRNA (Anzalone et al. 2020).

Since the discovery of the mechanism of interference in *Acidaminococcus* and *Lachnospiraceae* (Zetsche et al. 2015), CRISPR/Cas12a (formerly named CRISPR/Cpf1) has been adapted for genome editing. Unlike Cas9, Cas12a is guided by a single crRNA, and cleaves targeted DNA distal to T-rich PAM sequences, typically generating DSBs with 4-5-nt staggered overhangs (Fig. 1B). Cas12a orthologs from *Acidaminococcus* (AsCas12a), *Lachnospiraceae* (LbCas12a), and *Francisella novicida* (FnCas12a) have been studied intensively and are commonly used. Although CRISPR/Cas12a is thermosensitive, it is advantageous for multiplex editing (Zetsche et al. 2017); thus, it has become the second leading genome-editing tool.

Cas12b has been engineered to cleave both DNA strands (Strecker et al. 2019). Similar to Cas12a, Cas12b prefers T-rich PAMs. Unlike Cas12a, a sgRNA, engineered from a two-RNA structure (crRNA and tracrRNA), directs Cas12b to introduce a DSB with staggered ends (Fig. 1C). Cas12b orthologs have been successfully used for genome editing (Strecker et al. 2019; Ming et al. 2020; Wang et al. 2020a), and CRISPR/Cas12b has become the third most promising RNA-guided endonuclease platform.

Recently, other Cas12 nucleases have been explored for use in genome engineering. For example, Cas12e (formerly named CasX) and Cas12j (formerly named CasΦ) are active for eukaryotic genome modification (Liu et al. 2019; Pausch et al. 2020). Of particular interest is Cas12f (formerly named Cas14), which is less than half the size of Cas9/Cas12a. It allows robust gene editing and base editing in mammalian cells (Wu et al. 2021; Xu et al. 2021; Kim et al. 2022); thus, it may be useful in cell engineering and therapeutic applications.

### Engineered Cas nuclease variants

Expanding the target range is key to CRISPR/Cas technology development. Researchers have sought to develop engineered or evolved Cas9 or Cas12a variants with altered or relaxed PAM requirements. Several variants with less restrictive PAM compatibilities have been developed (Anzalone et al. 2020). Of particular interest is the near-PAMless SpCas9 variant, SpRY, which recognizes NRN (R = A or G) and NYN (Y = T or C) PAMs (NRN > NYN), and targets almost all PAMs (Walton et al. 2020). Together, these engineered Cas variants have substantially expanded the range of targets to include those that were previously inaccessible using CRISPR.

Improved specificity is another major priority in the development of CRISPR/Cas technologies. Researchers have developed several strategies to enhance the specificity, including exploring sgRNAs with a modified architecture, using dual nickase systems, rationally designing guide RNAs, transiently expressing editing reagents, and delivering editing reagents via preassembled Cas9:sgRNA ribonucleoprotein complexes (RNPs) (Anzalone et al. 2020; Li et al. 2019). Importantly, high-fidelity Cas variants have been rationally engineered or evolved. For example, based on structure-guided protein engineering, eSpCas9(1.1) (Slaymaker et al. 2016) and SpCas9-HF1 (Kleinstiver et al. 2016) have been developed to reduce off-target effects. Furthermore, using the same strategy, a high-fidelity Cas12a variant, enAsCas12a-HF1 (Kleinstiver et al. 2019), has been engineered to improve system specificity.

Although these Cas variants have expanded the target range and improved the specificity of CRISPR/Cas, the creation of robust Cas variants through protein engineering remains an important direction for the future advancement of CRISPR technologies. It is also worth to note that design sgRNAs through web tools (e.g., CRISPR-P, CRISPR-GE, CRISPR-PLANT v2, etc.) could reduce or avoid off-targeting (Lei et al. 2014; Minkenberg et al. 2019; Wang et al. 2020b; Xie et al. 2017).

### CRISPR-mediated base editing

CRISPR-mediated base editing enables direct, irreversible base conversions in a programmable manner, without creating DSBs. Current base editors are fusion proteins composed of catalytically impaired Cas nucleases and single-stranded DNA (ssDNA)-specific deaminases. Guided by guide RNAs, catalytically impaired Cas nucleases localize the ssDNA deaminase to the target sequence, forming a ssDNA R-loop (Anzalone et al. 2020). The nucleotides within the R-loop serve as substrates for the deaminase, and those nucleotide positions define the base editing ‘activity window’ (Komor et al. 2016). To date, two main classes of base editors have been developed: cytosine base editors (CBEs), which convert C–G to T–A base pairs, and adenine base editors (ABEs), which convert A–T to G–C base pairs (Komor et al. 2016; Gaudelli et al. 2017; Gürel et al. 2020).

**Fig. 2:**
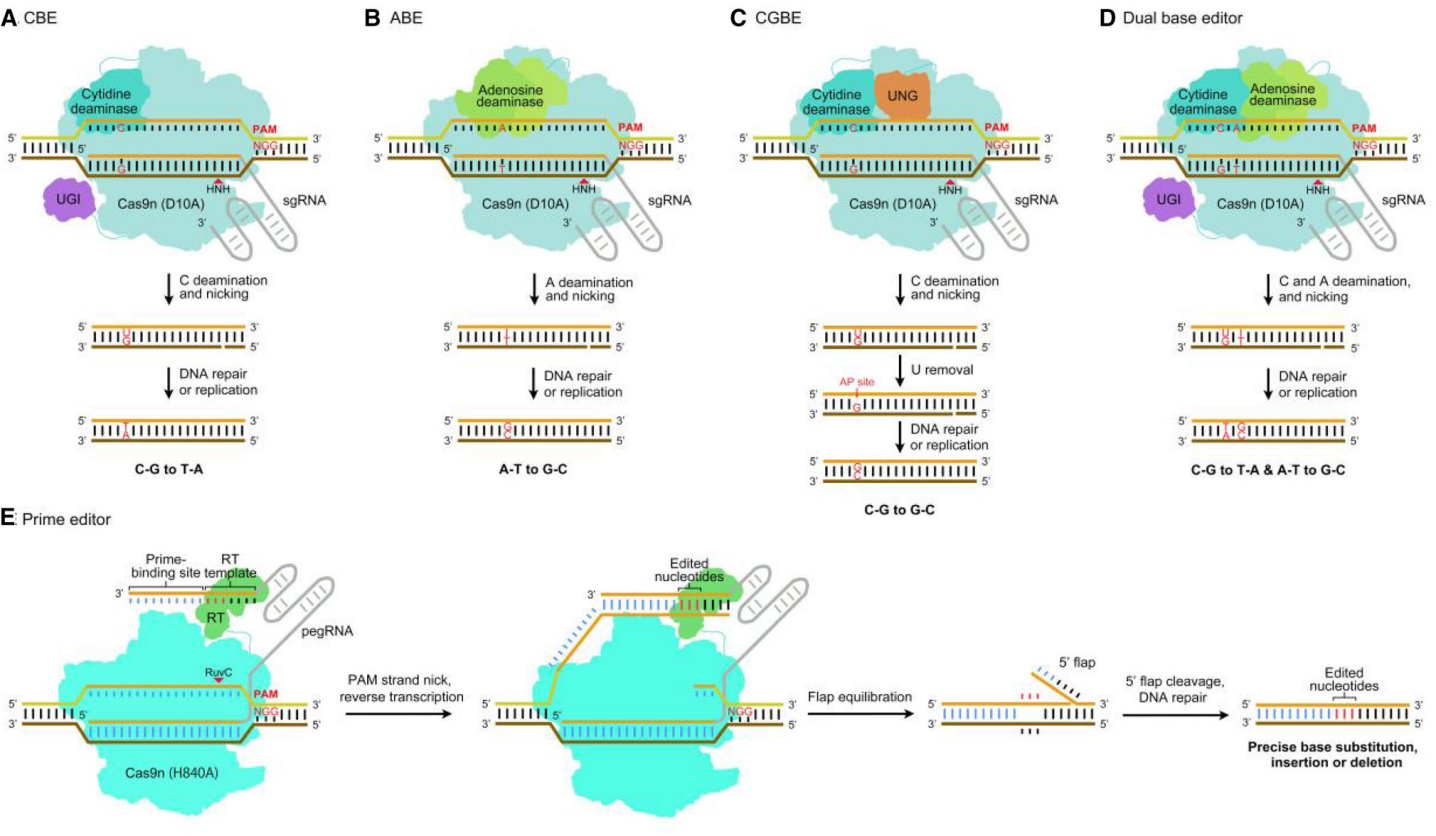
Illustration of CRISPR-mediated precise base editing. **A** A CBE-mediated C-to-T substitution. In CBEs, cytidine deaminase is used to directly deaminate cytidine (C) to uridine (U), resulting in a U–G mismatch. During DNA repair or replication, U is recognized as T, converting C–G to T–A base pairs. **B** An ABE-mediated A-to-G substitution. In ABEs, evolved deoxyadenosine deaminase is used to directly deaminate adenine (A) to inosine (I), which pairs with C, creating an A–T to G–C substitution. **C** A CGBE-mediated C-to-G substitution. In CGBEs, cytidine deaminase is used to deaminate C to U. Next, it is removed by UNG, creating an AP site. G is likely to be introduced into the site. This converts C–G to G–C base pairs during DNA repair or replication. **D** Dual base editor-mediated C-to-T and A-to-G substitutions. Cytidine and adenosine deaminases deaminate C to U and A to I, simultaneously. During DNA repair or replication, C-to-T and A-to-G substitutions are created. **E** Prime editor-mediated precise editing. Once the prime editor complex reaches the target sequence, Cas9n (H840A) nicks the strand. Then, the PBS binds to the 3' flap, and RT primes reverse transcription of the RT template with the desired mutations (red pegs). After equilibration, excision, and DNA repair, the edited nucleotides are precisely incorporated into the target site

In CBEs, cytidine deaminase is used to directly deaminate cytidine (C) within the ‘activity window’ to uridine (U), resulting in a U–G mismatch. During DNA repair or replication, U is recognized as T, converting C–G to T–A base pairs. Although efficient, targeted C-to-U conversions have been achieved in vitro, the efficiency of base editing in vivo is much lower (Komor et al. 2016). This is probably due to the cellular uridine base excision repair (BER) pathway. Uracil DNA glycosylase (UNG) removes U from DNA in cells and initiates the BER pathway, with reversion of the edited U–G back to a C–G pair. To subvert the BER pathway, uracil DNA glycosylase inhibitor (UGI) was fused to the C-terminus of the CBE architecture. This substantially increased the base editing efficiency. Therefore, CBEs typically include cytidine deaminase, Cas9 nickase, and UGI, and they can catalyze the conversion of C–G to T–A base pairs (Fig. 2A) in various cell types and organisms (Gao 2021).

Like cytosine, adenine contains an exocyclic amine that can be deaminated to yield inosine (I), which is read as guanosine (G) by polymerases. In theory, ABEs could be generated by replacing cytidine deaminase with adenine deaminase. However, there are no enzymes known to deaminate adenine in DNA. Therefore, scientists evolved *Escherichia coli* tRNA adenosine deaminase (TadA) into deoxyadenosine deaminase (TadA*), which can deaminate adenine on ssDNA. Next, TadA* was fused with Cas9 nickase to develop an ABE. To improve the editing efficiency, a wild-type TadA monomer was fused to the N-terminus of the ABE architecture. Among these ABEs, ABE7.10 is recommended for the conversion of A-T to G-C base pairs (Fig. 2B), and its effect has been verified in various cell types and organisms (Gaudelli et al. 2017). Subsequently, monomeric ABE8e has been used in human cells and a variety of species to augment the effectiveness and applicability of adenine base editing (Richter et al. 2020; Li et al. 2021d).

A cytosine transversion base editor (CGBE), which converts C to G in human cells and C to A in *E. coli* (Kurt et al. 2021; Zhao et al. 2021; Chen et al. 2021a), was developed. Structurally, the CGBE is similar to a CBE except that the CGBE contains UNG instead of UGI (Molla et al. 2020). In the CGBE, cytidine deaminase is used to deaminate C directly within the ‘activity window’ to U. Then, it is removed by UNG and creates an apurinic/apyrimidinic (AP) site, initiating the BER pathway. After Cas9 nickase nicks the non-edited strand, DNA repair and replication are activated, realizing the conversion of a C–G to a G–C base pair (Fig. 2C). Recently, the CGBE system has been established in rice, enabling efficient C-to-G editing (Tian et al. 2022). Thus, CGBE expands the base editing toolbox, and helps create new germplasm resources.

Dual base editor established by combining CBEs and ABEs has been developed in plants (Li et al. 2020b). It consists of cytidine deaminase, adenosine deaminase, Cas9 nickase, and UGI (Fig. 2D). Guided by a single sgRNA, the cytidine and adenosine deaminases deaminate C to U and A to I within the ‘activity window’, respectively. With DNA repair and replication, this creates C–G to T–A and A–T to G–C base pairs concurrently at the same target site. Dual base editors are valuable tools with broad potential applications, including facilitating the directed evolution of endogenous genes, accelerating the functional annotation of genomes, and aiding the development of therapies for genetic disorders.

### CRISPR-mediated prime editing

Recently, a powerful genome-editing technology named ‘prime editing’ was developed that can install all 12 possible types of base substitutions, small insertions and deletions, and even combinations of these edits (Anzalone et al. 2019). A prime editor consists of Cas9n (H840A) fused to reverse transcriptase (RT) and a prime editing guide RNA (pegRNA), which contains a prime-binding site (PBS) and a RT template at the extended 3' end of the sgRNA. Guided by the pegRNA, Cas9n (H840A) nicks the PAM-containing DNA strand and the PBS hybridizes with the newly liberated 3' end to form a prime-template complex. The RT domain then utilizes the 3' end of the nicked target DNA strand as a primer for reverse transcription. Therefore, the desired edit from the RT template can be permanently incorporated into the target site after excision of the redundant 5' flap and DNA repair of the non-edited strand (Fig. 2E).

As a versatile genome-editing tool, prime editing was initially reported to have limited efficiency. Therefore, various strategies have been used to improve its efficiency. For example, PE3 system, which utilizes an additional sgRNA to nick the non-edited strand, increases the editing efficiency ~ 3-fold. However, PE3 produces more undesired indels than PE2. PE3b, in which the added sgRNA targets only the edited sequence, provides a similar editing efficiency and fewer indel byproducts compared with PE3 (Anzalone et al. 2019). Transient co-expression of an engineered DNA mismatch repair (MMR) inhibiting protein has enhanced the editing efficiency. Similarly, in the absence of MMR, PE efficiency is enhanced 2–17-fold (Silva et al. 2022). PEmax systems, which have an optimized editor architecture, can enhance the editing efficiency by an average of 2.8-fold (Chen et al. 2021b). Engineered pegRNAs, with structural RNA motifs or the viral exoribonuclease-resistant RNA motif incorporated into the 3' terminus to enhance the stability, were found to improve the efficiency without increasing off-target editing activity (Nelson et al. 2022; Zhang et al. 2022). pegRNA, split into an sgRNA and a circular RNA RT template, exhibited comparable editing efficiency as that of the pegRNA (Liu et al. 2022). Moreover, various PAM-flexible Cas9 variants have been used in engineered prime editors, not only to increase the number of targetable sites but also to improve the prime editing efficiency (Kweon et al. 2021).

Prime editing has also been developed and tested in plants (Li et al. 2020c; Lu et al. 2021); however, the efficiency is limited. Intriguingly, raising the culture temperature to 37 °C increased the editing efficiency an average of 6.3% (Lin et al. 2020). In addition, optimization of the vector component through codon optimization for both Cas9 and M-MLV RT, improving the nuclear localization signal configuration using highly expressed endogenous promoters, and using an enhanced sgRNA scaffold and P2A self-cleaving peptides can improve the editing efficiency at some target sites up to 22-fold (Lu et al. 2021; Xu et al. 2020, 2021). In maize, enhancing pegRNA expression through two pegRNA cassettes or two promoter systems can increase the editing efficiency up to 53.2% (Jiang et al. 2020).

Design of the pegRNA is a major determinant of efficiency. For example, designing the PBS sequence with a melting temperature of 30 °C resulted in optimal editing efficiency; a paired-pegRNA strategy encoding the same edits on opposite DNA strands substantially enhanced the efficiency (Lin et al. 2021). Web-based tools (e.g., pegFinder, PlantPegDesigner, PE-Designer, PE-Analyzer, etc.) have been developed to simplify the design of pegRNAs and increase the PE efficiencies (Chow et al. 2021; Hwang et al. 2021; Lin et al. 2021). Recently, engineered Moloney-murine leukemia virus reverse transcriptase (e.g., removing ribonuclease H domain, incorporating a viral nucleocapsid protein, combining both methods) substantially improved prime editing efficiency (Zong et al. 2022). Overall, this revolutionary technology has great potential for plants provided that the editing efficiency is optimized.

## Current applications of CRISPR/Cas technologies in *Brassica*

CRISPR/Cas technologies can not only mutate a single gene but also mutate multiple genes simultaneously, generating stably inherited knockout mutants in major crops (Mao et al. 2019; Li et al. 2019). The application of CRISPR/Cas technologies to *Brassica* is rapidly increasing. Initially, marker/reporter genes such as *phytoene desaturase* were chosen as targets to establish CRISPR/Cas technologies. Since then, diverse endogenous genes have been targeted. Here, we summarize the application of CRISPR/Cas technologies in *Brassica* (Table 1).

**Table 1:** List of targeted genes and traits modified via CRISPR/Cas technologies in *Brassica*

Species	Targeted gene(s)	Editing methods	Repair results	Phenotype	Reference
*Brassica oleracea* var. capitata	*BolC.GA4.a*	CRISPR/Cas9	Indels	Dwarf, and affected pod valve margin	Lawrenson et al. (2015)
*Brassica oleracea* var. capitata	*BoPDS*, *BoSRK3*, *BoMS1*	CRISPR/Cas9, multiple gene editing	Indels	Albino phenotype, male sterility, self-compatibility	Ma et al. (2019a)
*Brassica oleracea* var. capitata	*BoMYB28*	CRISPR/Cas9	Indels	Reduced accumulation of methionine-derived glucosinolate	Neequaye et al. (2021)
*Brassica oleracea* var. capitata	*BoCER1*	CRISPR/Cas9	Indels	Reduced wax content, and brilliant green leaf	Cao et al. (2021)
*Brassica oleracea* var. capitata	*BoPDS*, *BoFRI*	CRISPR/Cas9, RNP	Indels	N.A	Murovec et al. (2018)
*Brassica oleracea* var. capitata	*BoGI*	CRISPR/Cas9, RNP	Indels	N.A	Park et al. (2019)
*Brassica oleracea* var. capitata	*BoPDS*	CRISPR/Cas9	Indels	Albino phenotype	Ma et al. (2019b)
*Brassica oleracea* var. capitata	*BoFAD2*	CRISPR/Cas9, double haploid inducer-mediated multiple gene editing	Indels	N.A	Li et al. (2021a)
*Brassica oleracea* var. *alboglabra*	*BoaCRTISO*	CRISPR/Cas9	Indels	Yellow leaves and bolting stems	Li et al. (2020a)
*Brassica rapa*	*BraPDS*, *BraFRI*	CRISPR/Cas9, RNP	Indels	N.A	Murovec et al. (2018)
*Brassica rapa*	*BraFLCs*	CRISPR/Cas9, multiple gene editing	Indels	Early-flowering	Jeong et al. (2019)
*Brassica rapa*	*BraHINS2*	CRISPR/Cas9	Indels	Etiolated and senesced at the cotyledon-seedling stage	Su et al. (2021)
*Brassica campestris*	Bra003491, Bra007665, Bra014410	CRISPR/Cas9, multiple gene editing	Indels	Methylation of pectin	Xiong et al. (2019)
*Brassica carinata*	*BcFLA1*	CRISPR/Cas9	Indels	N.A	Kirchner et al. (2017)
*Brassica napus* L	*BnALC*	CRISPR/Cas9	Indels	Increased shatter resistance	Braatz et al. (2017)
*Brassica napus* L	*BnMAX1*	CRISPR/Cas9, multiple gene editing	Indels	Decreased plant height, increased branch and silique numbers	Zheng et al. (2020)
*Brassica napus* L	*BnCLV3*	CRISPR/Cas9	Indels	Increased locule number of siliques	Yang et al. (2018)
*Brassica napus* L	*BnFAD2*	CRISPR/Cas9, double haploid inducer-mediated multiple gene editing	Indels	Increased oleic acid content	Okuzaki et al. (2018; Huang et al. (2020)
*Brassica napus* L	*BnTT8*	CRISPR/Cas9	Indels	Yellow-seeded traits	Zhai et al. (2020)
*Brassica napus* L	*BnTT2*	CRISPR/Cas9	Indels	Yellow-seeded traits	Xie et al. (2020)
*Brassica napus* L	*BnWRKY70*	CRISPR/Cas9	Indels	Enhanced resistance to *Sclerotinia*	Sun et al. (2018)
*Brassica napus* L	*BnGTR2*	CRISPR/Cas9, multiple gene editing	Indels	Small seeds with high oil content and low glucosinolates content	Tan et al. (2022)
*Brassica napus* L	*BnaA06.GTR2*	CRISPR/Cas9	Indels	Low seed glucosinolates content	He et al. (2022)
*Brassica napus* L	*BnRGA*, *BnFUL*, *BnDA1*, *BnDA2*	CRISPR/Cas9, multiple gene editing	Indels	Longer stems N.A	Yang et al. (2017)
*Brassica napus* L	*BnIND*	CRISPR/Cas9	Indels	Enhanced pod shatter resistance	Zhai et al. (2019)
*Brassica napus* L	*BnSPL3*	CRISPR/Cas9, multiple gene editing	Indels	Developmental delay phenotype	Li et al. (2018)
*Brassica napus* L	*BnSDG8*	CRISPR/Cas9	Indels	Early flowering	Jiang et al. (2018)
*Brassica napus* L	*BnLMI1*	CRISPR/Cas9	Indels	Unlobed leaves	Hu et al. (2018)
*Brassica napus* L	*BnJAG*	CRISPR/Cas9, multiple genome editing	Indels	Increased resistance to pod shattering	Zaman et al. (2019)
*Brassica napus* L	*BnLPAT2, BnLPAT5*	CRISPR/Cas9, multiple genome editing	Indels	Increased the size of oil bodies and decreased the oil content	Zhang et al. (2019)
*Brassica napus* L	*BnTFL1*	CRISPR/Cas9	Indels	Early flowering phenotype and altered plant architecture	Sriboon et al. (2020)
*Brassica napus* L	*BnA5.ZML1*	CRISPR/Cas9	Indels	Reduced self-compatibility	Duan et al. (2020)
*Brassica napus* L	*BnYCO*	CRISPR/Cas9, multiple genome editing	Indels	Yellow cotyledon and chlorotic true leaves	Liu et al. (2021)
*Brassica napus* L	*BnEOD3*	CRISPR/Cas9, multiple genome editing	Indels	Increased seed weight per plant	Khan et al. (2021)
*Brassica napus* L	*BnA9.WRKY47*	CRISPR/Cas9	Indels	Increased sensitivity to low boron	Feng et al. (2020)
*Brassica napus* L	*BnZEP*	CRISPR/Cas9, multiple gene editing	Indels	Orange-flowered rapeseed	Liu et al. (2020)
*Brassica napus* L	*BnMS5*	CRISPR/Cas9, multiple gene editing	Indels	Male sterility	Xin et al. (2020)
*Brassica napus* L	*BnD14*	CRISPR/Cas9, multiple gene editing	Indels	Prolific branching and dwarf	Stanic et al. (2021)
*Brassica napus* L	*BnaA03.BP*	CRISPR/Cas9, multiple gene editing	Indels	Semi-dwarf and compact architecture	Fan et al. (2021)
*Brassica napus* L	*BnS6-SMI2*	CRISPR/Cas9	Indels	Self-incompatibility	Dou et al. (2021)
*Brassica napus* L	*BnaSVP*	CRISPR/Cas9, multiple gene editing	Indels	Early-flowering phenotype	Ahmar et al. (2022)
*Brassica napus* L	*BnALS*	CBE	C to T	Herbicide-resistance	Wu et al. (2020)
*Brassica napus* L	*BnALS*, *BnRGA*, *BnIAA7*	CBE	C to T	Herbicide- resistance, semi-dwarf architecture	Cheng et al. (2021)
*Brassica oleracea var. botrytis*	*BoALS* *BoCENH3*	CBE	C to T	Herbicide-resistance N. A	Wang et al. (2022)
*Brassica napus* L	*BnFT*, *BnPDS*	ABE	A to G	N. A	Kang et al. (2018)

*Indels* insertions and deletions, *N.A.* not available

### Current applications of CRISPR/Cas technologies in *Brassica oleracea*

Cabbage (*Brassica oleracea* var. *capitata*) is an important leafy vegetable that is grown worldwide. In 2015, CRISPR/Cas9 technology was first applied to cabbage DH1012 by targeting *BolC.GA4.a*, an ortholog of *AtGA4*, which is involved in gibberellin biosynthesis. Cas9 driven by a constitutive promoter from Cassava Vein Mosaic Virus, and two sgRNAs targeting the first exon of *BolC.GA4.a* was constructed in the binary vector. Eighty independent transgenic lines were generated by *Agrobacterium*-mediated transformation. Through restriction digest/PCR assay, two lines with indels at the target sites were identified out of 20 T0 lines. Additionally, two lines with expected dwarf phenotype were identified through phenotypic screen. Homozygous mutants for *BolC.GA4.a* showed a dwarf phenotype, and the pod valve margin was affected (Lawrenson et al. 2015). This study demonstrated that CRISPR/Cas9 could induce targeted mutations in cabbage, and that the mutations could be stably transmitted across generations.

Due to gene redundancy, simply knocking out one gene may not cause a mutant phenotype. Thus, there is a need to develop tools with the ability to target multiple genes simultaneously. Based on endogenous tRNA processing, researchers developed a CRISPR/Cas9-mediated multiple gene editing system, which can target four sites in a single transformation, and the efficiencies range from 2.8% to 100%. It produced homozygous or biallelic mutations at multiple loci in the T0 generation (Ma et al. 2019a). This system provides an efficient and powerful tool to study gene function and improve traits in cabbage.

Since then, a number of studies on CRISPR/Cas9 applications have been published in cabbage. For example, the transcription factor MYB28, a key regulator of aliphatic glucosinolate (A-GSL) biosynthesis, was targeted using CRISPR/Cas9. The *myb28* mutant exhibited downregulated A-GSL biosynthesis gene expression and reduced accumulation of methionine-derived glucosinolate (Neequaye et al. 2021). Similarly, researchers obtained a stable *cer1* knockout line using CRISPR/Cas9. The genome-edited plant, which had a significantly reduced wax content, had brilliant green leaves (Cao et al. 2021). Together, these studies indicate that CRISPR/Cas9 technology is an important tool for functional genetic studies and the molecular breeding of cabbage.

### Current applications of CRISPR/Cas technologies in *Brassica rapa*

Chinese cabbage (*Brassica rapa* spp. *pekinensis*) is one of the most important vegetables in East Asia. Recently, genome editing was achieved in Chinese cabbage using CRISPR/Cas9 technology. Researchers developed a DNA-free method for the site-directed mutagenesis of endogenous genes using RNPs in protoplasts (Murovec et al. 2018). Meanwhile, *BraFLCs*, homologs of *AtFLC*, were targeted using CRISPR/Cas9. *Braflc2flc3* double-knockout lines were obtained, and the simultaneous mutations were stably inherited in the T1 generation. The edited plants showed an early-flowering phenotype that was independent of vernalization (Jeong et al. 2019), indicating that CRISPR can be used for molecular breeding.

Meanwhile, to confirm the role of *BraHINS2* in leaf yellowing, a vector targeting the first exon was constructed and used for transformation. Three T0 lines with targeted mutations were identified from thirteen independent transgenic plants. Mutants homozygous for *Brahins2* were obtained in the T1 generation; they were etiolated and senesced at the cotyledon-seedling stage (Su et al. 2021). This study demonstrates that CRISPR/Cas9 can be used to verify the functions of particular genes in Chinese cabbage.

### Current applications of CRISPR/Cas technologies in *Brassica napus*

Rapeseed (*Brassica napus* L.), an allopolyploid crop formed by hybridization between *B. oleracea* and *B. rapa*, is one of the most important oil crops worldwide. In 2017, CRISPR/Cas9 was applied to rapeseed. Two *BnALC* homoeologs were targeted knockout by a CRISPR/Cas9 construct containing only one target sequence, and T1 plants with four *alc* mutant alleles were obtained. All the mutations were faithfully transmitted to the T2 progeny. Siliques (5–6 cm long) from the *alc* mutants were more shatter-resistant than same-sized siliques of the cultivar (Braatz et al. 2017). This demonstrates the potential use of CRISPR/Cas9 for the simultaneous modification of genes in a polyploid species. As the applications of CRISPR/Cas9 technology in rapeseed mature, the targeted genes remain diverse but the main focus is on the genetic improvement of commercially important agronomic traits (e.g., yield, nutritional content, and stress resistance).

Yield improvement is a main goal of rapeseed breeding that can be increased using CRISPR/Cas9. For example, two sgRNAs were designed to target the two BnaMAX1 homologs. Various mutations were obtained in T0 generation, with the editing efficiency range from 56.30% to 67.38%. The mutations were passed on to the T1 generation. Targeted knockout of two *BnaMAX1* genes resulted in high-yield mutants with a significantly decreased plant height and increased branch and silique numbers (i.e., potential rapeseed ideotypes) (Zheng et al. 2020). Similarly, disrupting both copies of *BnaCLV3* increased the locule number in siliques, with a significantly higher number of seeds per silique and higher seed weight than in wild-type plants (Yang et al. 2018).

Improving the nutrient content of rapeseed is another important goal that can be achieved using CRISPR/Cas9. For example, knocking out all of the copies of *BnaFAD2* resulted in an increased oleic acid content in the mutant seeds (Okuzaki et al. 2018; Huang et al. 2020). Likewise, the targeted mutation of *BnaTT8* produced *tt8* mutants with yellow seeds that contained increased amounts of seed oil and protein, and an altered fatty acid composition (Zhai et al. 2020). Yellow-seeded mutants were also obtained by targeting both copies of *BnaTT2*; the resulting homozygous mutants exhibited an increased oil content and improved fatty acid composition (Xie et al. 2020).

In a final example, CRISPR/Cas9 was used to generate disease-resistant rapeseed plants by targeting WRKY transcription factors. Researchers constructed two vectors with multiple sgRNAs targeting two copies of *BnaWRKY11* and four copies of *BnaWRKY70*, respectively. The resulting *wrky70* mutants, but not the *wrky11* mutants, exhibited increased resistance to *Sclerotinia* (Sun et al. 2018).

It is worth to note that the phenotype of simultaneous editing multiple duplicated genes is inconsistent with that of editing a single gene sometimes. For example, knocking out all the multiple copies of *BnaGTR2* resulted in low seed glucosinolate mutants, however, these cannot be applied for rapeseed breeding because smaller seeds with increased seed oil content were observed (Tan et al. 2022). Very recently, *BnaA06.GTR2*, a crucial player in seed glucosinolate accumulation, has been targeted knockout. Low seed glucosinolate germplasms were obtained, and no negative effect on yield-related traits were observed (He et al. 2022). Together, CRISPR/Cas9 could not only target a single copy gene but also multi-copy genes, and play important roles for the application in polyploid rapeseed breeding.

Besides CRISPR/Cas9, base editors have been successfully applied to rapeseed. For instance, a CBE including rat cytidine deaminase was created that can precisely convert C to T within editing windows from positions 4 to 8 in the protospacer (Wu et al. 2020). Furthermore, a newly developed A3A-PBE system consisting of hAPOBEC3A cytidine deaminase was established. It was more efficient at generating C-to-T conversions within editing windows ranging from C1 to C10 in the protospacer, thus broadening the base-editing window in rapeseed (Cheng et al. 2021) *BnaALS* was precisely base-edited using a CBE, conferring herbicide resistance to rapeseed (Wu et al. 2020; Cheng et al. 2021). Very recently, CBE has been used to precisely edit ALS in cauliflower; mutants showed strong herbicide resistance (Wang et al. 2022). Another base editor, an ABE, has been applied to rapeseed protoplasts, leading to efficient A-to-G conversions (Kang et al. 2018). These experiments show that CRISPR/Cas technologies can be used to generate valuable germplasm resources for fundamental research on rapeseed and novel variety creation.

## Challenges and future perspectives

CRISPR/Cas technologies show specific, robust, multiplex genome-engineering capabilities. They have been widely used in plant genome editing and played important roles in creating various germplasm resources for crop breeding and biological research (Gao 2021). CRISPR/Cas9 has also been used to create desired mutants in polyploid *Brassica* species (Table 1). However, there are challenges to its comprehensive application.

### Genome complexity of *Brassica*

The genomes of *Brassica* species are complex, often with a high ploidy due to their long history of evolution and domestication. Though considerable effort has been made to elucidate the functions of many genes in *Brassica*, current progress in functional genomics lags far behind that in other crops. Fortunately, with the advent of advanced sequencing technologies and reduced sequencing costs, there has been a dramatic increase in the number of sequenced *Brassica* genomes (Chen et al. 2019a; Sun et al. 2022). It is easy to identify candidate genes controlling important agronomic traits through homology-based cloning (Karamat et al. 2021). CRISPR/Cas technologies enable multiplex genome editing; therefore, they offer a shortcut to link homologous genes to phenotypes.

### Establishing efficient and stable genetic transformation systems

Once a gene is selected for editing, genetic transformation is needed to deliver the CRISPR/Cas9 components into plant cells, followed by tissue culture and plant regeneration. Edited plants can then be screened from among the regenerated fertile plants. However, there is no well-established protocol for the genetic transformation of most *Brassica* crops. Nanotechnology has been proposed as a key driver to address delivery challenges and enhance the utility of plant genetic engineering (Cunningham et al. 2018). For example, nanomaterials enable the delivery of DNA into intact plants, with strong protein expression despite a lack of DNA integration (Demirer et al. 2019). Nanoparticles could potentially be used to deliver editing reagents to *Brassica* cells. Furthermore, recent studies have reported that overexpressing developmental regulators could improve the efficiency of plant regeneration from tissue culture in various transformation-recalcitrant species and genotypes (Lowe et al. 2016; Nelson-Vasilchik et al. 2018; Debernardi et al. 2020; Kong et al. 2020; Wang et al. 2022). These growth regulators could also be used to generate gene-edited dicots through de novo meristem induction (Maher et al. 2020). Additionally, RUBY has served as a visible and convenient selection marker for transformation in plants. Combining RUBY with CRISPR/Cas9 gene editing cassettes could facilitate to identify gene-edited and transgene-free plants (He et al. 2020). Moreover, a virus-induced genome-editing approach has been developed in wheat, bypassing tissue culture-based transformation (Li et al. 2021c). These strategies promise to expedite progress in genome editing in *Brassica* species.

*Agrobacterium*-mediated genetic transformation is the most commonly used approach to deliver CRISPR/Cas9 components into plant cells; however, it is restricted to particular genotypes. Recently, the discovery of transgenerational CRISPR/Cas9 activity has facilitated multiple gene editing in plants (Li et al. 2021b). Furthermore, several transformation-recalcitrant crops have been successfully modified using haploid inducer-mediated genome-editing systems (Kelliher et al. 2019; Wang et al. 2019; Budhagatapalli et al. 2020). Importantly, multiple gene homoeologs in *B. oleracea* and *B. napus* have been directly modified using a doubled haploid inducer-mediated genome-editing system (Li et al. 2021a). These systems, which enable genome editing in any elite commercial background and can produce transgene-free gene-edited crops when pollinated with (doubled) haploid inducer lines harboring CRISPR/Cas reagents, should be explored for use in transformation-recalcitrant *Brassica* crops and genotypes. Combined with interspecific hybridization programs, CRISPR/Cas9 will improve the agronomic traits of *Brassica* crops and accelerate breeding.

### Adopting precise and versatile genome-editing tools

Some valuable traits are conferred by single-nucleotide polymorphisms or defined insertions/deletions (Cheng et al. 2016). Thus, harnessing genetic diversity and modifying genomes precisely will be important for crop breeding programs. Until now, apart from the CBEs applied to rapeseed (Ahmar et al. 2022; Wu et al. 2020), precise genome editing (e.g., HDR-based gene targeting, ABEs, and prime editing) had not been achieved in *Brassica*, and most mutants were obtained via the NHEJ pathway. There is a need for the entry of scientists whose focus is on genome editing into the *Brassica* field to promote new technologies for precise genome editing. Recently, prime editing and prime editing-based technologies have been used to create point mutations, insertions, fragment deletions, replacements, and inversions (Anzalone et al. 2019, 2021; Choi et al. 2022; Jiang et al. 2022). It will be interesting to apply such versatile and precise technologies to *Brassica*. Moreover, the efficiency of prime editing systems varies remarkably by target site and cell type (Gao 2021). The DNA repair mechanisms that function in various cell states and cell types, as major determinants of editing efficiency, have not been fully elucidated (Anzalone et al. 2020). Additionally, other issues (e.g., excising redundant 5' flaps, repairing non-edited strands, and preventing byproduct generation) must be addressed. Therefore, effort should be made to optimize prime editing systems; they will facilitate *Brassica* breeding by modifying gene functions as desired, pyramiding multiple traits, or introducing elite alleles into predetermined safe-harbor loci.

Structural variations (SVs) have widespread impacts on the expression of nearby genes; thus, they play an important role in plant evolution and domestication (Alonge et al. 2020). For example, a *B. rapa* pan-genome was constructed using 18 genomes. Various SVs have been identified and genotyped, revealing the roles of SVs in intraspecific diversification and morphological domestication. Specifically, four SV-related genes are speculated to be involved in leaf-heading domestication (Cai et al. 2021). However, these SVs are not achievable using classical breeding. Chromosome structure engineering (e.g., inversions and translocations) has recently been achieved using CRISPR/Cas technology in plants (Schmidt et al. 2020). Similar systems should be established in *Brassica* to fix or break genetic linkages, providing huge potential for breeding new varieties with improved traits.

### Attitudes toward genome-edited crops

One roadblock to commercializing gene-edited crops is that the process involves genetic modification (Mao et al. 2019; Gao 2021). The current stringent and costly regulation of transgenic genetically modified crops is mainly due to the introduction of foreign DNAs. However, CRISPR/Cas technologies could improve crop traits by altering endogenous genes without transferring transgenes across species boundaries. This may allay fears associated with CRISPR-edited transgene-free plants, reducing the investment in time and money. Due to their low cost and versatility, CRISPR/Cas technologies have been used in various crops, including *Brassica*. With careful deployment and scientifically informed regulation, DNA-free genome-editing technologies will play important roles in crop improvement programs.

## Conclusions

The advent of facile, direct, and precise genome-editing tools using CRISPR/Cas9 has revolutionized plant biology research and crop breeding. Moreover, the expanding knowledge of CRISPR/Cas technologies will continue to be used for innovative applications, which promise to change the pace and course of agricultural research. However, CRISPR/Cas technologies should not be misunderstood as a panacea. Many other achievements are needed as well, including advances in basic genetic research, the development of novel delivery methods, increasing public confidence in the safety of CRISPRed crops, and developing conducive regulatory frameworks. We expect that CRISPR/Cas technologies will be fully applied in *Brassica*, facilitate the development of functional genomics, and help breed new *Brassica* varieties with improved agronomic traits.

## Data Availability

The datasets generated during and/or analyzed during the current study are available from the corresponding author on reasonable request.
